# Research on the impact of algorithmic trading on market volatility

**DOI:** 10.1038/s41598-025-15020-w

**Published:** 2025-08-17

**Authors:** Dan Yang, Yang Yang, Jia Luo, Zeming Wang, Haowei Sha

**Affiliations:** 1 School of Economics and Management, Chengdu Sport University, No. 1942, Huanhu North Road, Eastern New District, Chengdu, China; 2 School of Mathematics, Southwest University of Finance and Economics, No. 555, Liutai Avenue, Wenjiang District, Chengdu, 611130 China; 3 Business School, Chengdu University, No. 2025, Chengluo Avenue, Chengdu, China; 4 Finance Department of Leshan City Commercial Bank Corporate, No.423 Chunhua South Road, Central City District, Leshan, China

**Keywords:** Algorithmic trading, Market volatility, Financial market, Mediation model, Scientific data, Statistics

## Abstract

The rapid growing of algorithmic trading (AT) has been playing an increasingly important role in shaping financial market in recent years. Although many scholars have studied the impact of AT on market volatility, the evidence they provided is inconsistent. Whether and how AT influence on market volatility is still not clear, especially for emerging markets. In this paper, using level 2 quotations from Chinese market, we answered the question. By introducing multiple mediator model, we found that AT can significantly reduce market volatility. In addition to the influence of AT itself, the sentiment effect accounts for approximate 1/4 of the influence, and a very small portion, about 4%, of the influence can be explained by herd effect. Besides, it also illustrated that the influences of AT are more sensitive in main board, rather than in GEM board. Nevertheless, the sentiment effect has been playing plays more important role in the influence of AT in GEM board. These findings provide new insights into previous inconsistent evidence regarding the influence of AT on market volatility. They also have important implications for investment strategies and market regulations.

## Introduction

Algorithmic trading (AT) already accounts for a significant proportion of the total trading volume in some developed markets. The rapid growth of AT has raised concerns among investors, regulators and academia. One of the key research questions is whether AT is conducive to the healthy functioning of markets? Scholars in this field have first studied the impact of AT on market liquidity and volatility^1^, and they have consistently concluded that AT increases market liquidity [1–5]. However, there are still different conclusions regarding the impact of volatility. Some studies argue that AT reduces market volatility [6, 7]. Other studies contend that AT increases market volatility [5, 8, 9]. Among these studies, Boehmer et al. [5] attempt to discuss the mechanism by which AT influences volatility. In general, it is just beginning to investigate the influence of AT on market volatility, and the underlying mechanisms of this influence for scholars.

In this study, we have focused on the influence of AT on market volatility and aimed to understand how this influence was realized. According to traditional research, scholars often assume that investors are rational and will make investment decisions with the goal of maximizing expected utility. However, there is growing evidence that there are investors with bounded rationality in the market whose trading decisions are influenced by their own sentiment and beliefs and often deviate from the optimal choice [10, 11]. Therefore, investor sentiment is an important factor that influences investors’ decisions, and some irrational investment behaviors [12] may adversely affect the market. We endeavor to explain the mechanism by which AT influences volatility from the perspective of investor sentiment based on the following two facts. First, there is a substantial body of literature confirming that investor sentiment has a significant impact on market volatility [13–15]. The other fact is that AT can influence investor sentiment through multiple channels. This fact has not only been relatively overlooked in previous literature on AT, but also a more pronounced phenomenon in emerging markets like China, where retail investors play a key role. Based on previous studies, we believe that AT influences investor sentiment mainly in the following two ways. First, in some cases, AT directly replaces investors in decision-making, as they process market information, submit orders, and generate trade reports according to established algorithms. The influence of subjective sentiment on trading decisions is thus avoided, leading to a reduction in sentiment (irrational) trading in the market. Second, AT implies that stocks with higher levels of AT have more reasonable pricing by enhancing liquidity, reducing spread and improving information efficiency [16]. In addition to investor sentiment, we argued that the herd effect can be seemed as an alternative pathway through which AT influence on market volatility [17, 18]. In financial markets, large orders can stimulate herd effects because traders, especially individual investors who lack information, perceive new valuable information in the market and choose to follow large orders. However, AT reduces the number of large orders in trading and thus reduces the stimulus to investor sentiment. On the one hand, investors split their large orders through AT strategy to reduce execution costs. On the other hand, AT will profit from capturing large orders of other traders through speed advantage, which increases the execution costs of large orders of other traders [19] and leads to a decrease of large orders in the market.

In summary, based on the serial multiple mediation (SMM) model introduced by Hayes [20], we set investor sentiment as the first mediating variable and herd effect as the second mediating variable, and introduces the herd effect to analyze how AT influences volatility by influencing investor sentiment and investor behavior (referring to herd behavior in the present study), as well as the association between them. The sample data are drawn from the trading data of Shenzhen Stock Exchange (SZSE) in a down cycle and a market stabilization period, and we use the changes in the list of Shenzhen Connect (SC) underlying assets and order-to-trade ratios (OTRs) as instrumental variables because they are highly associated with AT. Besides, we consider them to be independent of volatility, although they may be associated with liquidity. Nevertheless, we control the liquidity indicators in the control items to exclude confounding factors.

The results of the present study show that (1) AT reduces stock price volatility. Specifically, for every 1 unit increase in the AT proxy variable, on average, the standard deviation of intraday returns will decrease by 0.817. (2) AT reduces spread and market depth. (3) It is found through mediating effect analysis that investor sentiment accounts for about 1/4 of the total effect and that AT reduces stock price volatility by reducing investor sentiment. (4) Only a small fraction (4%) of the effect is explained by the herd effect. AT reduces herd behavior in the market, thus reducing stock price volatility. (5) We find that in different groups AT mediates the influence of investor sentiment on the volatility of stocks listed on the GEM Board much more significantly than it does on the Main Board. Specifically, AT reduces more “bad” volatility generated by investor sentiment on the GEM Board, suggesting that currently AT is more valuable for the GEM Board. This has important implications for the formulation of AT-related policies. (6) When the market is in a period of decline, the role of AT in reducing volatility will decrease, while the mediating effect of AT on investor sentiment will also decrease, especially on the GEM Board. This indicates the sensitivity of the sentiment mechanism to the market environment.

The main contributions of the present paper lie in the following three aspects. First, our study overcomes data limitations and examines an emerging market that is rarely studied in existing literature. And it finds that AT reduces stock price volatility and improves market quality, complementing the evidence on related aspects. Second, our study explains the mechanism by which AT influences volatility from the perspective of investor sentiment, providing a deeper level of insight into understanding the role of AT. Third, we point out that AT is currently more valuable for GEM stocks, which has important implications for the formulation of AT-related policies.

The remainder of this paper is structured as follows; Section "Literature review" presents the literature review; Section "Algorithmic trading and market volatility" describes the data characteristics; Section "Investor behavior and market volatility" introduces the construction of variables and descriptive statistics; Section "Data" presents the effects of AT on liquidity and volatility; Section "Variables and descriptive statistics" further analyzes the mechanism by which AT influences volatility; and Section "AT measures" discusses the variation of the effect of different market conditions and the results of robustness tests.

## Literature review

### Algorithmic trading and market volatility

The method of using computers and algorithms to execute trading decisions is called algorithmic trading, which has become the mainstream mode in the financial market. On the one hand, algorithms can independently develop reasonable order submission strategies, determine the number, amount, and direction of orders submitted in each period, minimize the impact of large orders on the market and reduce transaction execution costs. For example, Chen [21] suggests that AT reduces the impact on the market through large order segmentation, which can effectively reduce transaction costs. Xia [22] indicates that the origin of algorithmic trading is to effectively reduce the market impact cost of large orders: the mother orders with large transaction volumes are automatically split by computer programs according to specific trading strategies and logic; after the split, smaller orders are automatically reported for trading at a fixed time and quantity, so as to obtain a better transaction price for the entire transaction. On the other hand, the automatic and rapid execution of AT may contribute to the sharp rise and fall, which is not conducive to the stable and healthy development of the securities market [23].

AT is believed not only facilitates trading, but also easily cause significant fluctuations in the securities market. However, as we mentioned before, the impact of AT on market volatility [5, 8, 9] increases as well as decreases [6, 7]. Therefore, it is necessary to reveal the impact mechanism of AT on market volatility.

## Investor behavior and market volatility

Unlike the assumptions about rational investors in traditional finance, in reality, it is difficult for investors to maintain complete rationality in decision-making, and investors have limited access to market information as well as the ability to analyze information, which often results in irrational investment behavior. With the rapid development of behavioral finance, the relationship between investor behavior and market volatility has received extensive attention from scholars. There are many factors influencing market volatility, such as the general economic situation, fiscal policy and monetary policy. These factors will eventually reach investors, who will receive, screen, and discriminate information, and form investment decisions that will drive trading behavior based on their established perceptions, investment experience, and investment preferences, and then influence market price fluctuations through collective investor behavior.

Shiller [24] argues that investors are influenced by their own perceptions and market expectations, which cause them to generate investor sentiment that in turn leads to irrational investment decisions. Baker et al. [25] finds that sentiment causes investors to blindly base their decision-making behavior on relevant information or popular opinion and to trade frequently, triggering herd behavior and increasing market volatility. Bouteska et al. [26] used the American Association of Individual Investors Index (AAII) as a proxy for investor sentiment and found that the linkage effect of stock index futures and spot decreases significantly as investor sentiment increases. Lee et al. [27] report that investor sentiment can drive stock market volatility and that either optimistic or pessimistic sentiment can easily create asset price bubbles, leading to dramatic price swings. In contrast to the findings of Lee et al. [27], the study carried out by Glasserman and Mamaysky [28] suggests that investor sentiment, as a systematic risk factor, has different influences on market volatility with negative sentiment leading to increased volatility and optimistic sentiment leading to decreased volatility. Sprenger et al. [29] find through Twitter posts that investor bullish sentiment is positively correlated with volatility and that higher posting volume is associated with higher market volatility. Da et al. [30] constructed a panic sentiment indicator reflecting the overall market and found that high panic leads to increased returns and increased market volatility. Mehra and Sah [31] point out that investor sentiment volatility is significantly correlated with stock price volatility based on a regression analysis of risk appetite parameters. Verma et al. [32] included individual and institutional investor sentiment linearly in an EGARCH model, and their study shows that investor sentiment has a significant effect on stock volatility for both individual and institutional investors. Uygur et al. [33] incorporated investor sentiment into a GARCH model and found that there is asymmetric volatility in the market. In other words, bad news is more likely to cause volatility than good news. Yang et al. [34] introduced investor sentiment into the HAR model and found a significant increase in the predictability of investor sentiment on market volatility.

Scholars have also analyzed the influence of investor behavior on market volatility from the perspective of herd behavior. Herd behavior refers to the behavior of investors who, after getting familiar with the behavior of existing traders in the market, give up their private information and choose to imitate the behavior of other investors [35]. Avramov et al. [36] propose that herd behavior increases volatility after a stock price decline, while informed trading decreases volatility after a stock price increase. Cai et al. [37] analyzed the influence of investors’ herd behavior on bond prices using U.S. corporate bond market data and found that buyers’ herd behavior will effectively help bonds realize their true value, while sellers’ herd behavior will distort asset prices. Compared to individual investors, institutional investors have more information advantage [38], more rational investment behavior [39], and lower risk preference [40]. As a result, the herd behavior of institutional investors can better stabilize the market compared to that of individual investors. Choi and Skiba [41] studied the herd behavior of institutional investors in international markets and found that institutional investors are more concentrated in markets with higher information transparency. Their herd behavior is driven by correlated signals from basic information, and price adjustments are faster in markets with more transparent information [42–44].

## Data

The sample period for our study includes two periods, respectively from June to October 2018 and from June to October 2019, a period of market decline and a period of market shock. (We wanted to use a period of market extremes as a comparison to a normal period. However, we only had access to data since 2017. Therefore, we chose from the data a period of market decline and a period of market shock in which the SSE Composite Index fell from 3,084.75 on June 1, 2018 to 2,602.78 on October 31, 2018, a decline of more than 15%. And within the period of market shocks, the stock index increased from 2,898.7 points on June 1, 2019 to 2,929.06 points on October 31, 2019.) The reason why the period of market decline in 2008 or other periods with more significant market decline are not chosen is that SZSE only started to provide order data in 2017. We choose the same month in two adjacent years to control the influences of market structure changes and different months as much as possible. We use data from the Tushare platform, and the Level 2 quotes provided by SZSE. The Tushare platform provides basic data, daily quotes and financial data for all listed Chinese stocks. We obtained basic data on all stocks through Tushare, including stock symbols, exchange codes, listing and delisting dates, as well as daily quotes, and financial data of all stocks in the sample period. Level 2 market data is a real-time market data service launched by Shanghai Stock Exchange (SSE) and SZSE, which includes high-frequency data such as 10 levels market depth, order queue, and filled orders. The difference is that the SZSE Level 2 market data additionally includes order data, which records the information of each order in the market, including order submission and cancellation. We obtained the daily 5 bid/ask orders and order data in the sample period from the Caifutong Data Center. (Caifutong Data Center (http://www.caifushuju.cn/) is a data service center that provides all Level 2 data for the China market.) The 5 bid/ask orders are composed of snapshots taken every 3 s during the trading day and consist of the ticker symbol, trading price, trade direction, trading volume, as well as the trading price and volume from 1 to 5 levels market depth. The order data contains every electronic message during the trading day while recording the securities code, order time, order quantity, order price, and order type (limit buy, limit sell, buy order cancellation, and sell order cancellation). Since SSE does not provide order data, our sample includes only stocks listed and traded on SZSE. We exclude the sample data of stocks with less than 500 trading records or less than 1000 orders in a single day. The final sample data consists of 349,828 pieces of data of 2117 stocks in 204 trading days.

## Variables and descriptive statistics

Table 1 provides definitions and descriptive statistics of AT, volatility, sentiment indicators, herd, and liquidity variables. The measures of these variables are described below:

**Table 1:** Definitions of variables and descriptive statistics

		Pooled	GEM	MBM	SME	Within
Variable	Description	Mean	Mean	Mean	Mean	SD
$AT_{{{\text{volume}}}}$	Negative of the day’s trading volume (unit: 100 yuan) divided by the number of electronic messages (AT proxy variable)	−60.511	−64.160	−58.438	−58.456	28.701
$AT_{trades}$	Number of the day’s electronic messages divided by the number of filled orders (AT proxy variable)	6.566	6.483	6.776	6.534	3.195
$Depth1$	Intraday mean of 1 level average depth (thousand lots)	0.855	0.373	1.925	0.737	3.972
$Depth5$	Intraday mean of 5 levels average depth (thousand lots)	5.340	2.341	11.949	4.635	23.206
$RQS$	Intraday mean of relative quote spread (basis point)	16.108	14.525	18.637	16.202	7.771
$1/P$	Reciprocal of the day’s closing price	0.123	0.094	0.164	0.126	0.085
$Vol$	Trading volume of the day (unit: 1 million yuan)	115.439	107.691	138.789	110.551	240.286
$Herd$	Individual stock herd indicator measured by LSV method	−9.332	−10.306	−8.351	−8.992	22.750
$Sentiment$	Daily individual stock sentiment indicator measured by PCA method	0.000	0.156	−0.198	−0.034	1.000
$Amplitude$	Intraday price amplitude (basis point)	386.175	422.601	335.523	380.283	226.245
$\left| {Pctchg} \right|$	Absolute value of the day’s returns (basis point)	203.744	225.129	176.082	199.273	200.806
$Volatility$	Standard deviation of intraday returns at different times (basis point)	82.646	91.441	71.162	80.859	54.299

## AT measures

As to AT measures, some studies were able to use trading data in the U.S. market with identity information about the trading firms. Cases include the studies conducted by Brogaard et al. [45] and Carrion [3], whose data allowed them to identify whether the buyer and seller were associated with 26 AT firms, which enabled them to distinguish AT among all trades. In our data, the orders are anonymous and do not contain information about the identity of the buyer and seller, which makes it impossible to distinguish which orders are generated by AT and which by traditional trades. We therefore use the same approach as that used by Hendershott et al. [1] and Boehmer et al. [5] to seek a proxy variable for AT. We define the AT’s proxy variable *AT*_volume_ as the negative of the trading volume (unit: 100 yuan) divided by the total number of electronic messages.

1 $${AT}_{volume}=\frac{{volume}_{i,t}}{{messages}_{i,t}}$$

where *i* represent a stock and *t* represents a trading day. ${volume}_{i,t}$ is the trading volume of Stock *i* on Day *t* (unit: 100 yuan), and ${messages}_{i,t}$ is the total number of electronic messages (including the submission and cancellation of orders) for Stock *i* on Day t.

Meanwhile, similar to the study conducted by Malceniece et al. [46], we define AT ’s another proxy variable ${AT}_{trades}$ as the ratio of the total number of electronic messages to the number of trades.

2 $${AT}_{trades}=\frac{{messages}_{i,t}}{{trades}_{i,t}}$$

where ${trades}_{i,t}$ is the number of trades for Stock *i* on Day *t*. It describes the ratio of the number of orders to the number of filled orders.

It worth mentioning that firstly, both of the proxies ${AT}_{volume}$ and ${AT}_{trades}$ measure AT from the marketing level. Because the issue focused on by this study is how AT influence market volatility and can be seen as a marketing level problem, we believe an widely used marketing level measurement is appropriate for this study. Secondly, ${AT}_{volume}$ and ${AT}_{trades}$ can be seen as proxy variables that measure AT from two facets. ${AT}_{volume}$ measures AT from the perspective of trading results. The intuition behind the proxy variable is that compared to trades based on fundamental analysis, AT leads to significantly more electronic messages. As a results, ${AT}_{volume}$ may decrease with AT. The other proxy variable, ${AT}_{trades}$, is a measurement of AT from perspective of trading behaviors. In stock market, a higher proportion of AT will rapidly increase the share of electronic messages in overall trading activity, thereby significantly increase ${AT}_{trades}$. Finally, according to previous studies [13], scholars have already illustrated that the proxy variables exhibit high sensitivity to algorithmic trading (AT) activity and have been widely used in research related to AT and financial market [47–49].

## Volatility measures

We use the standard deviation of intraday returns (basis point) as the first measure of volatility ${Volatility}_{i,t}$. In our high-frequency data, this variable provides a good picture of the intraday price volatility of stocks.

We also measure volatility in terms of $\left|{Pctchg}_{i,t}\right|$, the absolute value of the daily return, and ${Amplitude}_{i,t}$, the amplitude over the trading day.

3 $${Amplitude}_{i,t}=\frac{{High}_{i,t}-{Low}_{i,t}}{{Close}_{i,t}}$$

where ${High}_{i,t}$ denotes the highest price of the day, ${Low}_{i,t}$ the lowest price of the day, and ${Close}_{i,t}$ the closing price of the day.

## Sentiment measures

For the measure of investor sentiment, based on the method put forward by Baker and Wurgler [50] and the study by Fu et al. [11], we construct a sentiment variable for each stock using three indicators, namely price-to-earnings ratio (PE), turnover ratio (TR), and buy-sell imbalance (BSI) by means of principal component analysis.

P/E is the ratio of a company’s stock price to its earnings per share. Han and Li [51] argue that P/E is a factor of investor sentiment because it has higher levels in bull markets and lower levels in bear markets. Rahman and Shamsuddin [52] show that after controlling the effects of fundamentals, P/E typically increases as investor sentiment increases. Therefore, we use P/E as an indicator of stock sentiment.

TR is the ratio of stock trading volume to the total number of stocks in a given period to capture sentiment trading in the market. Baker and Wurgler [50] suggest that TR can be used as an index of sentiment. In general, a high TR indicates high trading demand from sentiment investors, destabilizing asset prices [51].

The BSI of an individual stock is the difference in bid-ask volume divided by the sum of bid-ask volume in a given period. Kumar and Lee [53] suggest that BSI can be used as an investor sentiment index. A positive (negative) BSI indicates a positive (negative) change in investor sentiment. Therefore, we use BSI as an indicator of stock sentiment.

4 $$BSI_{i,t} = (B_{i,t} - S_{i,t} )/(B_{i,t} + S_{i,t} )$$

where $B_{i,t}$ is the number of buys of Stock *i* in Period *t*, and $S_{i,t}$ is the number of sells of Stock *i* in Period *t*. Thus, the measure index of sentiment is obtained using principal component analysis.

5 $$Sentment_{i,t} = 0.500PE_{i,t} + 0.717TR_{i,t} + 0.557BSI_{i,t}$$

The coefficients of all three factors are positive, indicating that the selected factors have a positive influence on sentiment, which is consistent with expectations. The largest contribution of all factors is made by TR, implying that investor sentiment of individual stocks is largely related to investors’ intention to trade.

## Herds measures

As to the herds measures, we use the method proposed by Lakonishok et al. [54]. If investors follow each other in buying and selling stocks in the same period, then investors in this period will mostly tend to be buyers or sellers, thus exhibiting the herd effect. This method was originally used to compare the average tendency of funds to buy or sell a given stock at the same time with the average tendency of investors to buy or sell that stock at the same time under the assumption of independent decision making, thus indirectly measuring the degree of correlation between investors buying or selling a given stock. We define the herd effect for a stock on a given date as follows.

5 $$Herd_{i,t} = 10000(\left| {P_{i,t} - P_{t} } \right| - AF_{i,t} )$$

where

6 $$P_{i,t} = B_{i,t} /(B_{i,t} + S_{i,t} )$$

7 $$P_{t} = \sum\limits_{i = 1}^{M} {B_{i,t} /(\sum\limits_{i = 1}^{M} {B_{i,t} + \sum\limits_{i = 1}^{M} {S_{i,t} } } )}$$

8 $$AF_{i,t} = E\left| {P_{i,t} - P_{t} } \right|$$

$P_{i,t}$ is the proportion of investors who are net buyers of Stock *i* on Trading Day* t*, $B_{i,t}$ the number of investors who are net buyers of Stock *i* on Trading Day *t*, and $S_{i,t}$ the number of investors who are net sellers of Stock *i* on Trading Day* t*. $P_{t}$ denotes the expectation of $P_{i,t}$, namely the proportion of net purchases of all stocks by investors in the market on Trading Day *t*. $AF_{i,t}$ is an adjustment factor that represents the expected value of $\left| {P_{i,t} - P_{t} } \right|$ when the market is free from herd behavior. It shows that if there is no herd behavior in the market, $\left| {P_{i,t} - P_{t} } \right|$ may not be zero, and its expected value is related to the number of investors who trade in Period *t*. Thus, assuming that investors are independent of each other in their decisions, then $B_{i,t}$ obeys a binomial distribution of Parameter ($N_{i,t}$,$P_{t}$), where $N_{i,t} = B_{i,t} + S_{i,t}$, referring to the number of investors who traded Stock *i* in Period *t*. In this Equation, the probability of $B_{i,t} = k$ is:

9 $$P(B_{i,t} = k) = C_{{N_{i,t} }}^{k} p_{t}^{k} (1 - p_{t} )^{{N_{i,t} - k}}$$

Therefore,

10 $$AF_{i,t} = \sum\limits_{k = 0}^{{N_{i,t} }} {\left| {k/N_{i,t} - P_{t} } \right|} C_{{N_{i,t} }}^{k} p_{t}^{k} (1 - p_{t} )^{{N_{i,t - k} }}$$

The value of $Herd_{i,t}$ indicates the percentage basis point that the number of investors in a one-sided market is greater than expected for Stock *i* on Trading Day *t*. The greater the value of $Herd_{i,t}$, the greater the degree of herd behavior of investors.

## Liquidity measures

The liquidity is measured in two ways, the percentage basis point of intraday time-weighted relative quote difference,

11 $$RQS_{i,t} = \frac{1}{J}10000\sum\limits_{j = 1}^{J} {\frac{{2(Ask_{i,t,j} - Bid_{i,t,j} )}}{{Ask_{i,t,j} + Bid_{i,t,j} }}}$$

where $Ask_{i,t,j}$ is the seller’s best ask for Stock *i* in Period *j* on Day *t*, $Bid_{i,t,j}$ is the buyer’s best bid for Stock *i* in Period *j* on Day *t*. Another measure of liquidity is the market depth of the stock, including 1 level market depth and 5 levels market depth.

12 $$Depth1_{i,t} = \frac{1}{J}\sum\limits_{j = 1}^{J} {(AskVol_{i,t,j}^{1} + } BidVol_{i,t,j}^{1} )$$

13 $$Depth5_{i,t} = \frac{1}{J}\sum\limits_{j = 1}^{J} {\sum\limits_{d = 1}^{5} {(AskVol_{i,t,j}^{d} + } } BidVol_{i,t,j}^{d} )$$

where $AskVol_{i,t,j}^{d}$ is the volume of selling *d*, and $BidVol_{i,t,j}^{d}$ the volume of buying *d*.

We argue that the scaling factor used in (6) and (12) serves to convert the Relative Quote Spread into a standardized metric expressed in basis points (bp). In addition, compared with traditional Order Imbalance (OI) ratios, the herd model employs binomial distribution to statistically identify non-random trading patterns, enabling it to distinguish genuine herding from market-wide movements. This makes the herd measure particularly valuable for behavioral finance research. The model’s standardization (using basis points) further allows cross-stock comparability, unlike OI which is sensitive to absolute trading volumes. For practical applications, OI remains useful for preliminary screening of unusual trading days, whereas the herd model provides deeper behavioral insights when analyzing market microstructure or investor irrationality. As a results, we use it as our research model.

To build a multi-level capital market, there are multiple sectors in the Chinese stock market. Companies that meet the listing requirements of different sectors can choose to list in the corresponding sectors. The Main Board and the SME Board have more stringent listing requirements and higher standards for financial indicators. In contrast, the GEM Board has more relaxed listing conditions but also has a higher investor threshold. Companies listed on the Main Board are generally industry leaders. Although most of them have gone through a period of rapid growth and are in a mature stage of slow growth, they are preferred by investors due to the good liquidity of their shares. The SME Board has the same listing conditions as the Main Board. However, the liquidity of the SME Board stocks is smaller than that of Main Board stocks. Besides, the market value and industry status of companies listed on the SME Board are inferior to those listed on the Main Board. But in general, companies listed on the SME Board have stronger growth potential, a bigger room for market value growth, and the potential to develop into an industry leader in the future. The GEM Board has the most lenient listing conditions. Companies listed on the GEM Board are mainly technology companies, which have tremendous potential for growth but also inherently carry greater risks. We grouped our sample by stock market sectors rather than by market capitalization size [5] or trading volume [46], which would make our conclusions more meaningful. This is because policies are usually formulated for a particular stock market sector rather than for stocks with different market capitalization sizes and trading volumes. (For example, SZSE monitors unusual trading of GEM stocks. See http://www.szse.cn/disclosure/notice/general/t20200612_578383.html.)

Table 1 shows that the Main Board has a higher level of AT compared to the GEM Board and the SME Board, which implies that AT is more active in the more liquid large-cap stocks [46]. In terms of liquidity, the market depth of Main Board stocks is much higher than that of SME Board and GEM Board stocks, about 2.5 times that of SME Board stocks and 5 times that of GEM Board stocks. However, in terms of quote spread, Main Board stocks have higher relative quote spreads, implying poorer liquidity. We believe this is related to stock prices, with Main Board stocks being on average less expensive than SMB Board and GEM Board stocks (higher price reciprocal *1/P*), implying that the same bid-ask spread has a larger relative quote spread on Main Board stocks. In terms of volatility, all three volatility indicators show that the GEM Board has the highest volatility and that the Main Board market has the lowest volatility. Therefore, there is more active AT, better liquidity and lower volatility among the Main Board stocks. Conversely, there is less AT, less liquidity and higher volatility among the GEM Board stocks.

## Analysis of AT’s impact on market quality

To obtain a causal relationship between AT and market quality, we need to find a suitable instrumental variable for IV estimation. There are two important conditions for the selection of instrumental variables. One is correlation, which means the instrumental variable needs to be highly correlated with the explanatory variable AT. The other is exclusion restriction, which means the instrumental variable is not correlated with the residual term in the regression model. Several scholars have used changes in market structure to construct instrumental variables, such as Hendershott et al. [16], Boehmer et al. [5], and Malceniece et al. (Hendershott et al. [1] use the exogenous event of staggered introduction of auto quote on the New York Stock Exchange (NYSE) as an instrumental variable; Boehmer et al. [5] use the implementation of colocation services in different global markets as an exogenous event to set instrumental variables; and Malceniece et al. [46] use stock access to the Chi-X platform trading as an exogenous event-setting instrumental variable.) [46]. Our idea is similar in that we use stock entry into Shenzhen Connect (SC) as an instrumental variable. Shenzhen-Hong Kong Stock Connect is an interconnection mechanism launched at the end of 2016 following the launch of Shanghai-Hong Kong Stock Connect in 2014. It allows mainland and Hong Kong investors to trade the stocks listed on the other exchange within the prescribed range using their local securities firms or brokers, with the aim of promoting law-based regulation, market-oriented operation and international participation of China’s capital market. A stock becoming an underlying asset of SC means that some overseas institutional investors can participate in the trading of that stock through Hong Kong Exchanges and Clearing Limited (HKEX). Since AT is still under systematic research and preliminary experimentation in China [55], while it is relatively mature in other developed markets around the world, the SZSE underlying open to foreign investors has a higher level of AT. We use $SC_{i,t}$ as our instrumental variable that is equal to 1 if Security *i* is a SZSE underlying in Period *t*, and 0 otherwise.

**Fig. 1:**
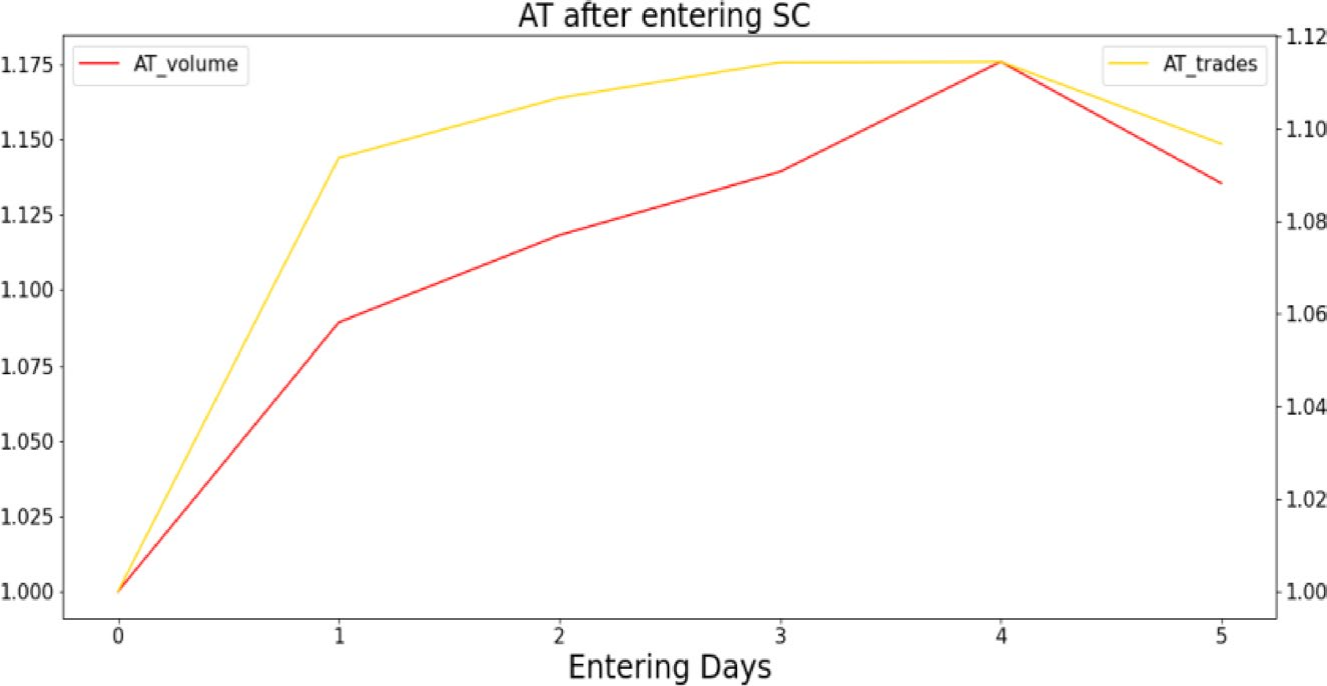
The change of AT after entering SC.

Figure 1 illustrates the correlation between the entry into SC list and the AT. The level of AT measured by both methods increased significantly over the five trading days, by approximately 10–15%. The most significant increase in AT level was observed on the first trading day after entry into SC, implying that entering SC accompany with higher level of AT activity. This indicate that the entering SC and AT activity is positive correlated.

In addition, the order to trade ratio (OTR) is also commonly used to measure AT activity level [14], which is highly correlated with AT activity intensity. (There is a huge correlation between OTR and AT, and especially between OTR and some HFT activities. This is because HFT generates a large number of orders, but only a few of them are actually filled. And most of them are withdrawn immediately after submission (Hasbrouck and Saar, [2]). To cut the number of orders generated by HFT, the Italian Stock Exchange adopted fines for high OTR (Rühl and Stein, 2014).) We construct a second instrumental variable and use order quantity and trading volume rather than order volume and filled orders to calculate $OTR_{i,t}$, which removes the effect of order size inconsistency and more accurately reflects the trades submitted during the day. The F-value of the weak instrumental variable test is much greater than 10, and it is concluded that there is no weak instrumental variable problem. The over-identification test p-value is 0.3290, which does not reject the original hypothesis. So, we consider the instrumental variable to satisfy exogeneity. Meanwhile, the test for AT endogeneity shows rejection of the original hypothesis, and we conclude that AT is endogenous.

Figure 1 depicts the change of AT level among stocks after entering SC, where the vertical coordinate indicates the comparison with the average AT level before entering SC, and the horizontal coordinate indicates the number of trading days after entering SC.

For causal analysis, we use two-stage least squares instrumental variables panel regressions. In the first stage, we regress the explanatory variable on the instrumental variable. We use a two-way fixed effects model to exclude time-invariant individual effects as well as time-invariant individual effects. Our control variables include the reciprocal of the stock price [8], liquidity indicators and trading volume. The regression equation for the first stage is.

14 $$AT_{i,t} = \alpha_{i} + \gamma_{t} + \varphi_{1} OTR_{i,t} + \varphi_{2} SC_{i,t} + \sum\nolimits_{j = 1}^{5} {\delta_{j} CV_{i,t}^{j} + \varepsilon_{i,t} }$$

In the first-stage regression, we generate fitted values of AT and use these values for the second-stage regression on the dependent variable. The regression equation is specified as follows:

15 $$Y_{i,t} = \alpha_{i} + \gamma_{t} + \beta_{0} \widehat{AT}_{i,t} + \sum\nolimits_{j = 1}^{5} {\delta_{j} CV_{i,t}^{j} } + \varepsilon_{i,t}$$

where $Y_{i,t}$ denotes one of the three volatility measures, including the standard deviation of intraday returns, absolute value of the day’s return and intraday amplitude. $\widehat{AT}_{i,t}$ denotes the fitted value of AT obtained from the first-stage regression. $CV_{i,t}$ stands for a set of control variables, including average 1 level depth $Depth1_{i,t}$, average 5 levels depth $Depth5_{i,t}$, price reciprocal $1/P_{i,t}$, relative quote spread $RQS_{i,t}$ and trading volume $Vol_{i,t}$. $\alpha_{i}$ and $\gamma_{i}$ denote individual and time fixed effects, respectively. We conduct the regression analysis for both the full sample and each subsample separately.

Table 2 shows the results of the second-stage regression. Except that AT has a non-significant influence on the absolute value of the day’s returns on the GEM Board, AT has a negative influence on different volatility indicators in all other groups, which means AT activity reduces the volatility of security prices. Our results are consistent with the results of the studies carried out by Hasbrouck and Saar [2] as well as Hagströmer and Nordén [7]. Our results show that for each unit increase in AT, on average, the standard deviation of intraday returns will decrease by 0.817, the absolute value of intraday return will decrease by 1.198 basis points, and the amplitude will decrease by 3.852 basis points. Among the different groups, AT has the strongest influence on the Main Board market, much higher than on the SMB Board and the GEM Board. One plausible explanation is that investors who adopt AT have limited attention capacity to follow real-time information of all stocks at the same time, while information related to Main Board stocks is more valuable in predicting future market movements. Besides, large-cap stock prices react to market information before small-cap stock prices [56]. Therefore, focusing on Main Board stocks is more beneficial for AT to take advantage of the new information to profit in the market. On the other hand, some of the more efficient AT, especially intraday AT usually do not hold positions overnight. However, due to the limitations of the A-share market trading system, they need to finance their positions with exchange-appointed brokerage firms to close out their positions. The brokerage firms set different margin ratios based on the risk of the underlying securities, and for low-risk Main Board stocks, the margin ratio will be lower than that of high-risk GEM stocks. Therefore, considering the cost of financing, AT prefers to be active in the Main Board market. Our results differ from the results of the study conducted by Malceniece et al. [46], who find that AT exerts a greater influence on small-cap stocks. The reason for the difference is that their study is targeted at developed markets, where the trading system and AT development are quite mature, and the increasing proportion and information processing capacity of AT makes it more valuable to focus on small-cap stock information. In contrast, in the Chinese market, AT is gradually emerging. It is still in a preliminary stage of development due to the trading system and technology, and it is mainly active among Main Board stocks.

**Table 2:** Second-stage regression of volatility on AT

		Control variables				
Sample	$\widehat{AT}_{i,t}$	$Depth1_{i,t}$	$Depth5_{i,t}$	$1/P_{i,t}$	$RQS_{i,t}$	$Vol_{i,t}$
Panel A: Impact of AT on $Volatility_{i,t}$						
Pooled	−0.817***	0.613*	−0.269***	−13.897	1.307***	0.058***
	(−12.91)	(1.95)	(−4.81)	(−0.66)	(15.90)	(5.77)
GEM	−0.621***	3.817***	−0.741***	−45.558	1.377***	0.099***
	(−6.40)	(2.91)	(−2.81)	(−1.45)	(11.35)	(4.24)
MBM	−1.205***	−0.615**	−0.075*	−25.385	1.418***	0.000
	(−12.31)	(−2.53)	(−1.70)	(−0.66)	(5.80)	(0.02)
SME	−0.764***	0.826	−0.365***	28.473	1.178***	0.074***
	(−7.64)	(1.37)	(−2.84)	(0.90)	(9.62)	(5.84)
Panel B: Impact of AT on $\left| {Pctchg} \right|_{i,t}$						
Pooled	−1.198***	11.514***	−1.939***	−123.384*	3.401***	0.262***
	(−5.50)	(4.05)	(−5.06)	(−1.69)	(11.35)	(8.08)
GEM	0.104	44.431***	−4.482***	−472.252***	3.870***	0.408***
	(0.33)	(4.27)	(−5.27)	(−4.21)	(9.45)	(5.39)
MBM	−3.288***	1.539	−0.570**	177.579	2.349**	0.043
	(−9.56)	(0.98)	(−2.46)	(1.29)	(2.46)	(1.09)
SME	−1.260***	18.255***	−2.854***	4.471	3.023***	0.301***
	(−3.32)	(4.01)	(−4.59)	(0.04)	(6.49)	(7.28)
Panel C: Impact of AT on $Amplitude_{i,t}$						
Pooled	−3.852***	−0.625	−0.633***	−376.945***	7.685***	0.282***
	(−11.41)	(−0.48)	(−2.81)	(−3.57)	(20.29)	(5.66)
GEM	−2.686***	4.626	−0.884	−558.808***	7.598***	0.485***
	(−6.06)	(1.48)	(−0.92)	(−3.70)	(14.10)	(4.29)
MBM	−5.820***	−4.593***	−0.069	−453.549**	8.838***	−0.003
	(−12.78)	(−4.71)	(−0.43)	(−2.38)	(7.40)	(−0.05)
SME	−3.636***	−2.008	−0.501	−211.237	7.494***	0.365***
	(−6.30)	(−0.95)	(−1.05)	(−1.28)	(12.74)	(5.59)

We also regress AT on liquidity. We measure liquidity in two ways. One is relative quote spread, which measures the spread between the best bid and ask prices in the market, and the smaller the spread, the higher the liquidity, and vice versa. The other is market depth, which measures the volume at different quotes and measures liquidity from another perspective, where higher depth is associated with higher liquidity, and vice versa. The regression results are reported in Table 3, where AT reduces relative quote spread, implying that AT increases liquidity. This result is consistent with the findings by scholars such as Hasbrouck and Saar [2] and Brogaard et al. [45]. However, when depth is used as a liquidity indicator, AT has a significant negative influence on both 1 level depth and 5 levels depth. We argue that spread and depth measure liquidity in two ways, with spread primarily capturing the price element of liquidity (width) and depth capturing the quantitative element of liquidity. As Brogaard [6] points out that AT provides the best bids and best asks for most of the trading day and avoids informed traders with certain strategies, but it provides only a quarter of the depth of non-AT. In fact, our results imply that AT non only profits by spread capturing, which leads to a reduction in spread in the market, but also reduces the depth of the market. This suggests that AT plays more of a liquidity demander role.

**Table 3:** Second-stage regression of liquidity on AT

		Control variables	
Sample	$\widehat{AT}_{i,t}$	$1/P_{i,t}$	$Vol_{i,t}$
Panel A: Impact of AT on $RQS_{i,t}$			
Pooled	−0.016***	86.502***	−0.001***
	(−3.69)	(62.94)	(−4.18)
GEM	−0.000	78.099***	−0.000
	(−0.05)	(28.38)	(−0.63)
MBM	−0.020***	92.444***	−0.001***
	(−4.37)	(35.93)	(−3.67)
SME	−0.028***	89.947***	−0.002***
	(−3.70)	(44.25)	(−4.04)
Panel B: Impact of AT on $Depth1_{i,t}$			
Pooled	−0.063***	20.323***	−0.003**
	(−2.64)	(4.89)	(−2.34)
GEM	−0.007***	7.546***	0.000
	(−2.60)	(3.16)	(0.58)
MBM	−0.216**	48.782***	−0.010**
	(−2.24)	(2.91)	(−2.26)
SME	−0.033***	17.178***	−0.001**
	(−3.54)	(5.94)	(−2.38)
Panel C: Impact of AT on $Depth5_{i,t}$			
Pooled	−0.186**	79.939***	−0.006**
	(−2.25)	(5.27)	(−2.01)
GEM	−0.024***	45.478***	0.001*
	(−2.62)	(3.11)	(1.95)
MBM	−0.643*	152.062**	−0.027*
	(−1.81)	(2.54)	(−1.91)
SME	−0.099***	78.056***	−0.002*
	(−3.69)	(5.99)	(−1.66)

## Analysis of the channels by which AT impacts volatility

In past empirical studies, the conclusions on the impact of AT on volatility are inconsistent [7, 46], with some studies suggesting that AT increases volatility in the market, while other literature suggesting the opposite. Regardless of the conclusions, these studies rarely answer why AT has such an influence on volatility. As many studies have found that AT improves liquidity and information efficiency, it is therefore natural for Boehmer et al. [5] to associate the influence of AT on volatility with the aforementioned effect. Therefore, they believe that the influence of AT on volatility may be due to improvements in liquidity and information efficiency. They control liquidity and information efficiency in their model, yet AT still has an effect on volatility, implying that it is ungrounded to attribute the effect of AT on volatility to liquidity and information efficiency.

Our study finds that AT reduces volatility. Furthermore, we are interested in the mechanism by which AT reduces volatility. We consider two mediators in our model, investor sentiment and investor behavior (represented by herds). On the one hand, a growing literature has noticed the role of investor sentiment and investor behavior on financial markets [11, 12], while no literature has yet mentioned whether AT interacts with investor sentiment and investor behavior. On the other hand, in an emerging market like China, there are a lot of individual investors who have more pronounced sentiment behavior characteristics [54]. We use the SMM model proposed by Hayes [20], which differs from the general mediation model in that it considers the association between mediators and is suitable for our exploration of the link between investor sentiment and investor behavior. Malceniece et al. [46] use this model to investigate the mediating role played by liquidity and delay in the mechanism by which AT influences comovement in returns. Similarly, we attempt to investigate the mechanism by which AT influences volatility through estimation of this model.

**Fig. 2:**
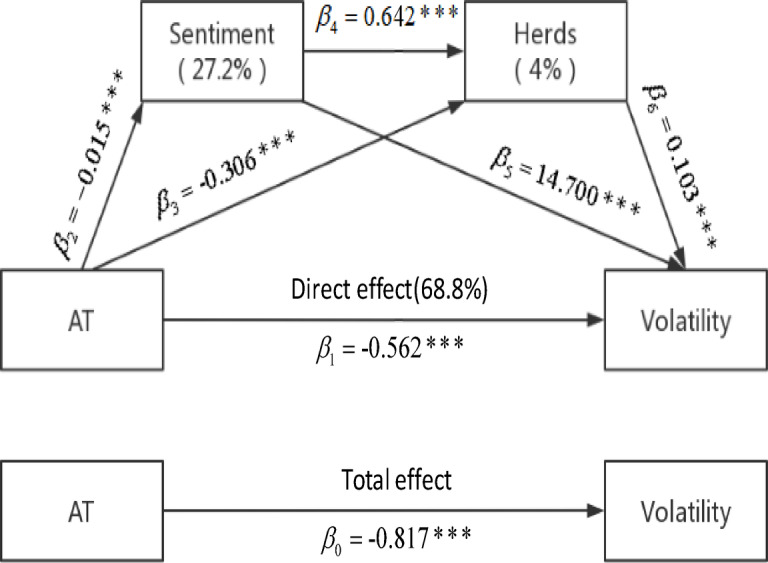
_Serial multiple mediation model of AT on Volatility._

Figure 2 shows the results obtained from the regression of the entire sample data. Hayes [20] identifies four paths of influence, which we divide here into three mechanisms by which AT influences volatility. The first is that AT reduces volatility by reducing investor sentiment, which corresponds to $\beta_{2} \beta_{5}$ in Fig. 2, and this mechanism accounts for about 1/4 of the total effect. There are three main reasons why AT reduces investor sentiment. First, a portion of AT is set by the algorithm to receive and process information in the market, identify existing trading opportunities in the market, and submit orders automatically. The entire process is done entirely by the algorithm without investor intervention, thus avoiding investors bringing sentiment into their trading decisions. Second, AT brings better liquidity and information efficiency, correcting misprice in the market in real time, and disrupting the positive feedback mechanism of sentiment trading. (Hirshleifer et al. [10] introduce this mechanism, where irrational traders drive stock prices up and rising prices cause investor sentiment to rise, further increasing their desire to buy, while rapid price increases attract new irrational investors, leading to a cycle of rising prices and sentiment.) In addition, large orders have a stimulating effect on investor sentiment, as other traders, especially individual investors who lack information, perceive the appearance of large orders as a sign of new information in the market and choose to follow them. And AT reduces the large orders in the market by splitting large orders through proxy algorithmic trading on the one hand. On the other hand, some AT profit from capturing large orders of other traders through speed advantage, increasing other traders’ execution cost of large orders [46], leading to splitting and hiding of orders by other traders. A large number of studies [54, 57] illustrate the positive association between investor sentiment and volatility, and one of the mechanisms of this association is irrational behavior of investors, such as “chasing noise” [54].

The second mechanism is that AT reduces volatility by reducing herd behavior in the market, which accounts for a small part of (only 4%) the total effect. This mechanism consists of two paths, AT increases—sentiment decreases—herd behavior decreases—volatility decreases, which corresponds to $\beta_{2} \beta_{4} \beta_{6}$ in Fig. 2. The first path is the stimulating effect of investor sentiment on investor behavior [43], where AT reduces the herd behavior triggered by sentiment by decreasing investor sentiment. In contrast, the effect of the second path is much larger than that of the first one ($\beta_{3} > > \beta_{2} \beta_{4}$), and we attribute the second path to the “reputational herding” among fund managers identified by Graham [58]. This refers to the fact that fund managers with a high reputation and salary choose to imitate other fund managers to keep their current salary. As AT evolves, the complexity and flexibility of investment strategies will increase, meaning that it will be more difficult for fund managers to imitate each other, thereby reducing this herding. Meanwhile, a higher degree of herding leads to greater market volatility [12], while AT reduces herding and thereby lowers volatility.

The third mechanism is the direct effect of AT, which corresponds to $\beta_{1}$ in Fig. 2, occupying about 70% of the total effect. Proxy algorithmic trading in AT is usually used to split large orders. When traders trade on a larger scale in the financial market, the larger single trade has a strong impact on the relatively illiquid market, which can cause instantaneous and violent market fluctuations. To reduce the negative impact of market volatility on trading, traders usually split the orders that need to be traded. In other words, they split large trades into several smaller trades and disperse them at the right time, thus reducing the execution cost and execution gap of the trades [1, 59]. Thus, we take the direct effect to correspond to the proxy algorithmic trading by splitting large orders and thus reducing volatility in the market. The direct effect accounts for a larger proportion of the total effect, implying that unlike in the U.S. market where high-frequency trading dominates, the current Chinese market is more dominated by proxy algorithmic trading.

The figure depicts the mechanisms by which AT influences volatility through investor sentiment and herds and the influence of each mechanism. The estimated values of the coefficients in the figure are obtained from regressions of the following equations.

16 $$Volatility_{i,t} = \alpha_{i} + \gamma_{t} + \beta_{0} \widehat{AT}_{i,t} + \sum\nolimits_{j = 1}^{5} {\delta_{j} CV_{i,t}^{j} } + \varepsilon_{i,t}$$

17 $$Sentiment_{i,t} = \alpha_{i} + \gamma_{t} + \beta_{2} \widehat{AT}_{i,t} + \sum\nolimits_{j = 1}^{5} {\delta_{j} CV_{i,t}^{j} } + \varepsilon_{i,t}$$

18 $$Herds_{i,t} = \alpha_{i} + \gamma_{t} + \beta_{3} \widehat{AT}_{i,t} + \beta_{4} Sentiment_{i,t} + \sum\nolimits_{j = 1}^{5} {\delta_{j} CV_{i,t}^{j} } + \varepsilon_{i,t}$$

19 $$Volatility_{i,t} = \alpha_{i} + \gamma_{t} + \beta_{1} \widehat{AT}_{i,t} + \beta_{5} Sentiment_{i,t} + \beta_{6} Herds_{i,t} + \sum\nolimits_{j = 1}^{5} {\delta_{j} CV_{i,t}^{j} } + \varepsilon_{i,t}$$

where $Volatility_{i,t}$ refers to the standard deviation of intraday returns, $Sentiment_{i,t}$ the sentiment variable calculated by the PCA-based method, $Herds_{i,t}$ the herd variable calculated by the LSV-based method, and $\widehat{AT}_{i,t}$ the fitted value of AT obtained from the first-stage regression. $CV_{i,t}^{{}}$ denotes a series of control variables, including average 1 level depth $Depth1_{i,t}$, average 5 levels depth $Depth5_{i,t}$, price reciprocal $1/P_{i,t}$, relative quote spread $RQS_{i,t}$, trading volume $Vol_{i,t}$, as well as individual and time fixed effects $\alpha_{i}$ and $\gamma_{i}$. Percentage indicates the proportion of mediating effect to the total effect. *, ** and *** represent 10%, 5% and 1% significance levels. The sample consists of 2117 stocks traded on SZSE, and the sample period includes two periods, respectively from June 1 to October 31, 2018 and from June 1 to October 31, 2018, adding up to a total of 10 months. The sample consists of observations made in 49,828 trading days.

Table 4 presents the parameter estimation results of the SMM model, including the regression results on the whole sample data and the different stock market sectors. Among the results for different groups, the results are in the same direction as the regression results for the whole sample data, except for the negative coefficient of sentiment on herds in the Main Board market. This does not affect our conclusion that the effect of AT on herds is mainly contributed by the direct effect and a small percentage through the sentiment-mediated effect ($\beta_{3} > > \beta_{2} \beta_{4}$) and that overall, AT still reduces the herds in the market. We calculate the contribution of each mechanism in different groups and find that the proportion of each mechanism varies dramatically across different stock market sectors. Specifically, in the GEM Board market, the mediating effect of investor sentiment contributes about 40% of the total effect, while in the Main Board market, the share is only 11%, and in the SME Board market, it is an intermediate value of 24%. This means that AT plays a bigger role in the GEM Board market by influencing investor sentiment to reduce volatility. As we discussed earlier, investor sentiment increases volatility and the mechanism behind this is that a series of irrational behaviors driven by sentiment increase short- and medium-term volatility in the market. This volatility adversely affects the market and investors, and we consider it a type of “bad” volatility compared to the “good” volatility generated by improved liquidity and information efficiency as pointed out by Boehmer et al. [5]. The implication of this finding is that although AT reduces volatility to a greater extent in the Main Board market, only a small fraction of volatility is the “bad” volatility caused by sentiment. In contrast, in the GEM Board market, AT reduces “bad” volatility to a greater extent, maintaining market stability. Therefore, we believe that AT is currently more valuable to the GEM Board, which has important implications for how AT-related policies should be formulated.

**Table 4:** Parameter estimation for the serial multiple mediation model

				Control variables				
Sample	$\widehat{AT}_{i,t}$	$Sentiment_{i,t}$	$Herds_{i,t}$	$Depth1_{i,t}$	$Depth5_{i,t}$	$1/P_{i,t}$	$RQS_{i,t}$	$Vol_{i,t}$
Panel A: Impact of AT on $Sentiment_{i,t}$								
Pooled	−0.015***			−0.010	0.002	0.106	−0.005***	0.001***
	(−12.08)			(−1.15)	(0.72)	(0.25)	(−3.33)	(5.14)
GEM	−0.017***			−0.010	0.016***	−0.569	−−0.008***	0.002***
	(−7.29)			(−0.88)	(3.98)	(−0.80)	(−3.44)	(3.63)
MBM	−0.011***			−0.014***	0.002	−1.572**	0.004	0.000
	(−7.20)			(−2.80)	(1.17)	(−2.57)	(1.61)	(0.77)
SME	−0.013***			−0.024***	0.010***	−0.020	−0.004**	0.001***
	(−6.99)			(−3.67)	(3.76)	(−0.03)	(−2.03)	(5.35)
Panel B: Impact of AT and $Sentiment_{i,t}$ on $Herds_{i,t}$								
Pooled	−0.306***	0.642***		−0.216***	0.027**	30.938***	−0.090***	−0.011***
	(−17.67)	(9.21)		(−6.79)	(2.35)	(7.05)	(−5.70)	(−11.60)
GEM	−0.253***	1.026***		−0.116	0.123***	14.304**	−0.100***	−0.008***
	(−10.95)	(7.99)		(−0.73)	(2.84)	(2.03)	(−4.18)	(−4.82)
MBM	−0.319***	−0.268**		−0.290***	0.028***	28.643***	−0.027	−0.013***
	(−10.65)	(−2.13)		(−6.87)	(3.81)	(3.10)	(−0.58)	(−8.74)
SME	−0.336***	0.445***		−0.195***	0.056***	34.455***	−0.088***	−0.012***
	(−10.56)	(4.21)		(−3.21)	(2.88)	(4.71)	(−3.68)	(−6.90)
Panel C: Impact of AT, $Sentiment_{i,t}$ and $Herds_{i,t}$ on $Volatility_{i,t}$								
Pooled	−0.562***	14.700***	0.103***	0.776***	−0.306***	−18.649	1.386***	0.043***
	(−11.94)	(22.80)	(35.93)	(2.96)	(−4.14)	(−1.07)	(16.59)	(5.73)
GEM	−0.343***	14.584***	0.096***	3.977***	−0.994***	−38.575	1.505***	0.070***
	(−5.38)	(10.86)	(20.77)	(3.20)	(−3.48)	(−1.43)	(12.32)	(3.92)
MBM	−1.036***	11.709***	0.111***	−0.421**	−0.098**	−10.216	1.371***	0.000
	(−11.73)	(6.79)	(17.36)	(−2.01)	(−2.05)	(−0.28)	(5.69)	(0.01)
SME	−0.544***	14.272***	0.103***	1.184**	−0.513***	25.196	1.238***	0.055***
	(−7.22)	(18.85)	(22.99)	(2.16)	(−3.91)	(0.98)	(9.75)	(5.59)

## Additional results and robustness tests

This section reports additional data results as well as partial results of the robustness tests. Section 7.1 shows the differences in the impact of AT on liquidity and volatility under different market conditions. Section 7.2 contains robustness tests performed by two methods, one with substitution of AT measures and volatility measures and the other with adjustment of control variables.

## The effects of AT in different market conditions

Several studies point out that the impact of AT on the market may be related to the market conditions [1, 46, 59] and that when market conditions become extreme, AT may choose to stop their strategies or use some shorting strategies to exacerbate the market volatility and worsen liquidity [9]. Our sample data include two periods, a period of market declines and a period of market shocks. We are interested in how the impact of AT changes across market conditions, and how the contribution of each mechanism changes. Therefore, we add a time dummy variable $D_{t}$ and cross terms of explanatory variables to Models (23)–(26), and our models become Eqs. (27)–(30).

20 $$Volatility_{i,t} = \alpha_{i} + \gamma_{t} + \beta_{0} \widehat{AT}_{i,t} + \phi_{0} \widehat{AT}_{i,t} D_{t} + \sum\nolimits_{j = 1}^{5} {\delta_{j} CV_{i,t}^{j} } + \sum\nolimits_{j = 1}^{5} {\chi_{j} CV_{i,t}^{j} } D_{t} + \varepsilon_{i,t}$$

21 $$Sentiment_{i,t} = \alpha_{i} + \gamma_{t} + \beta_{2} \widehat{AT}_{i,t} + \phi_{2} \widehat{AT}_{i,t} D_{t} + \sum\nolimits_{j = 1}^{5} {\delta_{j} CV_{i,t}^{j} } + \sum\nolimits_{j = 1}^{5} {\chi_{j} CV_{i,t}^{j} } D_{t} + \varepsilon_{i,t}$$

22 $$\begin{gathered} Herds_{i,t} = \alpha_{i} + \gamma_{t} + \beta_{3} \widehat{AT}_{i,t} + \phi_{3} \widehat{AT}_{i,t} D_{t} + \beta_{4} Sentiment_{i,t} + \phi_{4} Sentiment_{i,t} D_{t} \\ + \sum\nolimits_{j = 1}^{5} {\delta_{j} CV_{i,t}^{j} } + \sum\nolimits_{j = 1}^{5} {\chi_{j} CV_{i,t}^{j} } D_{t} + \varepsilon_{i,t} \\ \end{gathered}$$

23 $$\begin{gathered} Volatility_{i,t} = \alpha_{i} + \gamma_{t} + \beta_{1} \widehat{AT}_{i,t} + \phi_{1} \widehat{AT}_{i,t} D_{t} + \beta_{5} Sentiment_{i,t} + \phi_{5} Sentiment_{i,t} D_{t} \\ + \beta_{6} Herds_{i,t} + \phi_{6} Herds_{i,t} D_{t} + \sum\nolimits_{j = 1}^{5} {\delta_{j} CV_{i,t}^{j} } + \sum\nolimits_{j = 1}^{5} {\chi_{j} CV_{i,t}^{j} } D_{t} + \varepsilon_{i,t} \\ \end{gathered}$$

where $Volatility_{i,t}$ denotes the standard deviation of intraday returns, $\widehat{AT}_{i,t}$ the fitted value of AT obtained from the first-stage regression, and $D_{t}$ a time dummy variable that takes the value of 1 in periods of market decline (2018) and 0 in other periods (2019). $CV_{i,t}$ denotes a set of control variables, including average 1 level depth $Depth1_{i,t}$, average 5 levels depth $Depth5_{i,t}$, price reciprocal $1/P_{i,t}$, relative quote spread $RQS_{i,t}$, trading volume $Vol_{i,t}$, as well as individual and time fixed effects $\alpha_{i}$ and $\gamma_{i}$. $\phi$ represents the difference in impact across market conditions. Since time fixed effects are already considered, it is not necessary to include $D_{t}$ in the model. We run regressions for the entire sample data and for each group respectively.

Table 5 reports the impact of AT under different market conditions. First, we find that the volatility-reducing effect of AT is discounted during periods of market decline. Although after further grouping, this effect is only significant on the SME Board, it still implies that AT is sensitive to market conditions. When the market signals risk, AT may choose to exit the market [9]. Second, the effect of AT on sentiment and herds also declines in periods of market decline (except on the Main Board where the effect of AT on herds is not statistically significantly different in the two periods). Seen from the contribution of the different mechanisms, the mediating effect of sentiment and herds decreases while the direct effect increases, especially on the GEM Board. As we discussed earlier, AT is more valuable for the GEM Board as it reduces more “bad” volatility caused by sentiment. Yet at the same time, we find this effect to be fragile. The contribution of the sentiment mechanism in the GEM Board decreases significantly when market conditions become worse. On the one hand, AT reduces sentiment dependent on information efficiency, which is reduced by the fact that companies may disclose less unfavorable information in a declining market to maintain share price stability. GEM stocks receive less attention and regulation, meaning that information is less transparent. On the other hand, the role of AT in reducing sentiment by reducing large orders can also fail in periods of declining markets because when large order traders need to sell their stocks, they may simply dump their orders directly in the market rather than splitting them.

**Table 5:** Serial multiple mediation models under different market conditions

	$\widehat{AT}_{i,t}$		$Sentiment_{i,t}$		$Herds_{i,t}$	
Sample	$\beta$	$\phi$	$\beta$	$\phi$	$\beta$	$\phi$
Panel A: Impact of AT on $Volatility_{i,t}$						
Pooled	−0.941***	0.181*				
	(−12.89)	(1.76)				
GEM	−0.677***	0.034				
	(−4.31)	(0.19)				
MBM	−1.176***	−0.055				
	(−9.01)	(−0.32)				
SME	−0.996***	0.371***				
	(−9.43)	(2.59)				
Panel B: Impact of AT on $Sentiment_{i,t}$						
Pooled	−0.025***	0.015***				
	(−17.17)	(8.48)				
GEM	−0.029***	0.019***				
	(−9.89)	(5.61)				
MBM	−0.017***	0.009**				
	(−5.29)	(2.56)				
SME	−0.022***	0.014***				
	(−12.18)	(6.08)				
Panel C: Impact of AT and Sentiment on $Herds_{i,t}$						
Pooled	−0.398***	0.145***	0.550***	−0.017		
	(−20.30)	(5.03)	(6.91)	(−0.18)		
GEM	−0.330***	0.122***	1.013***	−0.215		
	(−10.45)	(2.96)	(8.13)	(−1.36)		
MBM	−0.372***	0.091	−0.502***	0.352*		
	(−7.87)	(1.44)	(−2.95)	(1.65)		
SME	−0.470***	0.200***	0.338***	−0.089		
	(−17.33)	(4.32)	(2.93)	(−0.66)		
Panel D: Impact of AT,$Sentiment_{i,t}$ and $Herds_{i,t}$ on $Volatility_{i,t}$						
Pooled	−0.476***	−0.154*	16.659***	−4.877***	0.113***	−0.021***
	(−7.48)	(−1.72)	(19.39)	(−4.56)	(29.47)	(−3.62)
GEM	−0.107	−0.412***	17.640***	−7.331***	0.117***	−0.039***
	(−0.94)	(−2.90)	(11.59)	(−4.60)	(17.29)	(−3.59)
MBM	−0.924***	−0.205	12.779***	−2.402	0.103***	0.012
	(−7.64)	(−1.22)	(5.40)	(−0.71)	(13.34)	(1.09)
SME	−0.584***	0.064	15.967***	−5.026***	0.110***	−0.018**
	(−6.14)	(0.50)	(16.56)	(−4.64)	(18.65)	(−2.03)

## Robustness tests

To test the robustness of our results, we adjust the model in two ways. The first is replacing the proxy variables for AT and volatility, and the second by adjusting our control variables.

We re-regress Eqs. (18)–(21) using another proxy variable for AT $AT_{trades}$ constructed by Malceniece et al. [46]. Specifically, it is the ratio of the total number of electronic messages to the number of transactions. Table 6 reports the corresponding regression results, and Fig. 2 illustrates the corresponding SMM model.

**Table 6:** Robustness test after substituting AT proxy variables

				Control variables				
Sample	$\widehat{AT}_{i,t}$	$Sentiment_{i,t}$	$Herds_{i,t}$	$Depth1_{i,t}$	$Depth5_{i,t}$	$1/P_{i,t}$	$RQS_{i,t}$	$Vol_{i,t}$
Panel A: Impact of AT on $Volatility_{i,t}$								
Pooled	−18.511***			3.243***	−0.552***	−403.758***	1.790***	0.290***
	(−12.92)			(9.85)	(−10.12)	(−13.89)	(20.45)	(21.02)
GEM	−14.087***			5.819***	−0.953***	−342.227***	1.743***	0.275***
	(−6.41)			(4.23)	(−3.61)	(−6.21)	(13.56)	(15.18)
MBM	−27.253***			3.258***	−0.491***	−599.643***	2.125***	0.342***
	(−12.28)			(11.54)	(−11.36)	(−12.09)	(8.48)	(15.45)
SME	−17.350***			3.288***	−0.628***	−336.498***	1.632***	0.292***
	(−7.64)			(5.23)	(−4.90)	(−9.23)	(12.50)	(13.20)
Panel B: Impact of AT on $Sentiment_{i,t}$								
Pooled	−0.336***			0.038***	−0.003	−7.023***	0.004***	0.005***
	(−11.98)			(4.27)	(−0.89)	(−12.08)	(2.67)	(19.13)
GEM	−0.384***			0.045***	0.011**	−8.718***	0.002	0.007***
	(−7.22)			(2.97)	(2.55)	(−6.81)	(0.76)	(14.61)
MBM	−0.258***			0.023***	−0.002	−7.020***	0.011***	0.003***
	(−7.07)			(3.75)	(−1.53)	(−8.00)	(4.00)	(8.92)
SME	−0.288***			0.017***	0.006**	−6.136***	0.004**	0.005***
	(−6.94)			(2.58)	(2.13)	(−8.66)	(2.01)	(12.11)
Panel C: Impact of AT and $Sentiment_{i,t}$ on $Herds_{i,t}$								
Pooled	−6.959***	0.646***		0.771***	−0.079***	−115.307***	0.091***	0.076***
	(−17.65)	(9.27)		(15.84)	(−6.25)	(−20.11)	(4.95)	(18.34)
GEM	−5.789***	1.029***		0.706***	0.036	−107.101***	0.051*	0.065***
	(−10.95)	(8.02)		(4.01)	(0.81)	(−12.23)	(1.89)	(11.39)
MBM	−7.215***	−0.267**		0.736***	−0.082***	−123.412***	0.161***	0.078***
	(−10.62)	(−2.11)		(10.26)	(−8.17)	(−12.26)	(3.41)	(10.85)
SME	−7.642***	0.449***		0.889***	−0.060***	−126.176***	0.111***	0.084***
	(−10.55)	(4.25)		(9.53)	(−2.81)	(−12.67)	(3.80)	(11.06)
Panel D: Impact of AT, $Sentiment_{i,t}$ and $Herds_{i,t}$ on $Volatility_{i,t}$								
Pooled	−12.834***	14.706***	0.103***	2.595***	−0.501***	−288.112***	1.720***	0.204***
	(−11.96)	(22.80)	(35.88)	(9.53)	(−6.83)	(−13.10)	(19.81)	(17.45)
GEM	−7.897***	14.587***	0.096***	5.098***	−1.113***	−203.937***	1.710***	0.168***
	(−5.40)	(10.86)	(20.75)	(4.02)	(−3.90)	(−5.67)	(13.40)	(9.12)
MBM	−23.436***	11.713***	0.111***	2.909***	−0.456***	−503.894***	1.979***	0.294***
	(−11.70)	(6.79)	(17.36)	(11.79)	(−9.68)	(−10.91)	(8.04)	(14.24)
SME	−12.433***	14.276***	0.103***	2.945***	−0.702***	−235.608***	1.563***	0.211***
	(−7.24)	(18.86)	(22.97)	(5.13)	(−5.31)	(−7.94)	(11.89)	(11.70)

Our results are robust in terms of the sign of the regression coefficients as well as their significance. AT still has a significantly negative effect on volatility, both overall and after dividing the market into sectors, and the effect is more pronounced in the Main Board market, much more than in the GEM market. Through the regression of the SMM model, the various mechanisms by which AT influences volatility remain significant. The contribution of each mechanism does not change significantly, where the share of investor sentiment decreases by 0.5%, corresponding to a 0.5% increase in the share of direct effects, while the share of herds remains unchanged. Across different sector markets, the GEM market still has a more pronounced sentiment effect, implying that AT is more likely to reduce stock price volatility by reducing investor sentiment.

**Fig. 3:**
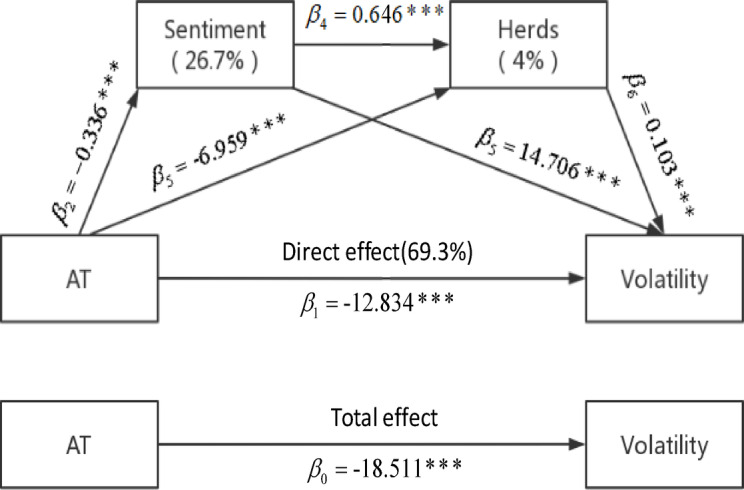
Mediating and direct effects of substituting AT proxy variables on volatility.

Figure 3 depicts the mechanisms by which AT influences volatility through investor sentiment and herds and the impact of each mechanism. The estimated values of the coefficients in the figure are obtained from the following model regressions:

25 $$Sentiment_{i,t} = \alpha_{i} + \gamma_{t} + \beta_{2} AT_{i,t} + \sum\nolimits_{j = 1}^{5} {\delta_{j} CV_{i,t}^{j} } + \varepsilon_{i,t}$$

26 $$Herds_{i,t} = \alpha_{i} + \gamma_{t} + \beta_{3} \widehat{AT}_{i,t} + \beta_{4} Sentiment_{i,t} + \sum\nolimits_{j = 1}^{5} {\delta_{j} CV_{i,t}^{j} } + \varepsilon_{i,t}$$

27 $$Volatility_{i,t} = \alpha_{i} + \gamma_{t} + \beta_{1} \widehat{AT}_{i,t} + \beta_{5} Sentiment_{i,t} + \beta_{6} Herds_{i,t} + \sum\nolimits_{j = 1}^{5} {\delta_{j} CV_{i,t}^{j} } + \varepsilon_{i,t}$$

where $Volatility_{i,t}$ is the standard deviation of intraday returns, $Sentiment_{i,t}$ denotes the sentiment variable calculated by the PCA-based method, $Herds_{i,t}$ denotes the herds variable calculated by the LSV-based method, and $\widehat{AT}_{i,t}$ denotes the fitted value of AT obtained from the first-stage regression. $CV_{i,t}^{{}}$ denotes a series of control variables, including average 1 level depth $Depth1_{i,t}$, average 5 levels depth $Depth5_{i,t}$, price reciprocal $1/P_{i,t}$, relative quote spread $RQS_{i,t}$, trading volume $Vol_{i,t}$, as well as individual and time fixed effects $\alpha_{i}$ and $\gamma_{i}$. Percentage indicates the proportion of mediating effect to the total effect. *, **, and *** represent 10%, 5%, and 1% significance levels. The sample includes 2117 stocks traded on SZSE, and the sample period includes two periods, respectively from June 1 to October 31, 2018 and from June 1 to October 31, 2019, adding up to a total of 10 months. The sample consists of observations made in a total of 349,828 trading days.

In addition, it worth mention that more proxy variables of AT are chosen by us to replicate the research in order to ensure the robustness of the conclusions. For example, the proxy variable used by Hindershott et al. [47], Yan et al. [60], and Chen [61] are all used by use to replicate our study. These replications of the study illustrate that the conclusions of our study have exhivited strong robustness and borad adaptability.

## Conclusion

Based on the Level 2 order data provided by SZSE, we measured AT activity in the Chinese securities market. We also analyzed the causal relationship between AT and liquidity as well as that between AT and volatility using the change in the SC underlying list and OTRs as our instrumental variables. Furthermore, we analyzed the mechanisms by which AT influences stock price volatility through an SMM model, with our first mediator being investor sentiment and the second mediator being the herd effect. We introduced the herd effect to illustrate how AT influences volatility by influencing investor sentiment and investor behavior (which in our study refers to the herd effect) as well as the association between them.

Our results show that AT reduces stock price volatility, and our findings are robust using other volatility indicators such as the absolute value of daily return or intraday amplitude. In addition, we also find that AT reduces spread and market depth. Through the mediating effect analysis, our results show that the sentiment effect accounts for about 1/4 of the total effect and that AT’s trade automation, correction of mispricing and reduction of large orders lead to lower investor sentiment, which results in reduced stock price volatility. A very small portion (4%) of the effect is explained by the herd effect, where AT reduces stock price volatility by reducing herd behavior caused by sentiment and “reputation”. Among the different groups, we find a much larger share of the mediating effect of AT on volatility through investor sentiment on the GEM Board than on the Main Board, implying that AT reduces a larger share of “bad” volatility generated by investor sentiment on the GEM Board. This suggests that AT is currently more valuable for the GEM Board. However, we also find that this effect is fragile, and when the market is in a period of decline, the role played by the sentiment mechanism will be discounted.

Our study contributes to existing literature in the following three ways. First, we overcome data limitations to study an emerging market that is rarely covered in exiting literature, and we find that AT reduces stock price volatility and improves market quality, adding to the evidence on related aspects. Second, we explain the mechanisms by which AT influences volatility from the perspective of investor sentiment, providing a deeper level of insight into understanding the role of AT. Finally, we point out that AT is currently more valuable for GEM stocks, which has important implications for the formulation of AT-related policies.

Building on this study, several promising research directions emerge: First, the interaction effects between AT and different types of institutional investors (e.g., hedge funds vs. mutual funds) warrant investigation, particularly how their trading strategies may amplify or mitigate AT’s impact on volatility. Second, the development of more sophisticated sentiment indicators incorporating alternative data sources (e.g., social media analytics, news sentiment) could provide deeper insights into the behavioral mechanisms. Third, comparative studies across emerging markets with varying levels of AT penetration would help establish boundary conditions for our findings. Lastly, the evolving regulatory landscape calls for longitudinal studies to assess how policy changes moderate AT’s effects on market quality, especially during periods of financial stress.

## Data Availability

Data is provided within the manuscript， if all the datas are needed, the corresponding author will provide.
